# Association between chest pain and serum total bilirubin levels in adults: Cross-sectional analysis of NHANES 2005 to 2018

**DOI:** 10.1097/MD.0000000000050772

**Published:** 2026-10-23

**Authors:** Zhenjie Fan, Zhiyong Wang, Jun You, Yiwen He, Yan Cheng, Jiaxue Tang, Qian Min

**Affiliations:** a The First College of Clinical Medical Science, China Three Gorges University, Yichang, China; b Department of Emergency, Yichang Central People’s Hospital (Xiling Campus), Yichang, China.

**Keywords:** bilirubin, cardiovascular disease, chest pain, obesity

## Abstract

The association between serum total bilirubin (STB) and chest pain remains unclear. This study aimed to explore the potential relationship between STB and the prevalence of chest pain. Data from the National Health and Nutrition Examination Survey 2005 to 2018 were analyzed to evaluate the association between STB levels and chest pain. Logistic regression (odds ratio with 95% confidence interval), restricted cubic spline curves, and stratified subgroup analyses were performed, and a total of 19,187 participants were enrolled. STB was negatively correlated with chest pain; each unit increment in log_2_(STB) corresponded to a 9% reduction in the risk of chest pain (odds ratio = 0.91, 95% confidence interval: 0.85–0.96, *P* < .001). The restricted cubic spline curve revealed a nonlinear relationship between log_2_(STB) and chest pain (*P* for nonlinearity = .008), with an inflection point at 14.42 μmol/L. The association between STB and chest pain was consistent across multiple subgroups. Mildly elevated STB concentrations were associated with a lower prevalence of chest pain in our study population, suggesting that STB may have potential value for risk stratification of chest pain. Nevertheless, additional prospective studies are required to verify the causal relationship between them.

## 1. Introduction

Chest pain is defined as discomfort and/or a sense of oppression originating from the sternum and its surrounding areas. In the United States, chest pain ranks as the second leading cause of emergency department visits.^[1]^ Each year, more than 8 million patients with chest pain receive outpatient evaluation, and approximately 1-quarter of patients presenting with chest pain require hospital admission for observation.^[2,3]^ Chest pain has an extensive differential diagnosis covering respiratory, musculoskeletal, psychological, gastric, and esophageal disorders, among which cardiovascular diseases are the most life-threatening, including coronary heart disease (CHD), hypertension, angina pectoris, and heart failure. Cardiovascular diseases carry fatal potential risks, and chest pain serves as the most typical warning sign, especially for CHD. Meanwhile, the occurrence of chest pain can predict cardiovascular events.^[4]^ Moreover, chest pain exacerbates the burden of depression, and a bidirectional relationship exists between depression and chest pain, which severely impairs physical function and quality of life in patients with chest pain.^[5,6]^ Therefore, identifying influencing factors for chest pain is of great clinical significance.

Serum total bilirubin (STB) is the final product of heme catabolism by heme oxygenase during erythrocyte senescence and is one of the most potent endogenous antioxidants, which has been confirmed to be inversely associated with the risks of various diseases. Mildly elevated STB correlates with lower cardiovascular disease risk, particularly coronary artery disease. Meanwhile, STB exerts potential protective effects against cardiovascular risk factors, including nonalcoholic fatty liver disease,^[7]^ stroke,^[8]^ peripheral arterial disease,^[9]^ diabetes mellitus, and its complications (retinopathy, microalbuminuria).^[10–12]^ Moreover, multiple studies have found relationships between bilirubin and atrial fibrillation,^[13]^ pulmonary embolism,^[14]^ herpes zoster,^[15]^ anxiety, and depression. It is widely recognized that chest pain is a common symptom of these diseases.

In summary, the biological functions of bilirubin have been gradually elucidated, and regulating bilirubin levels to prevent and treat chronic diseases has become a popular research topic, which includes multiple disorders that can induce chest pain. On this basis, we hypothesized that STB, which mirrors systemic inflammatory and oxidative stress burden, is significantly associated with the risk of developing chest pain. The present study was designed to verify this potential relationship, compare the incidence of chest pain across populations with varying STB concentrations, and furnish evidence supporting clinical prevention, treatment, and health management for chest pain.

## 2. Materials and methods

### 2.1. Ethical approval declarations

The National Health and Nutrition Examination Survey (NHANES) is conducted by the Centers for Disease Control and Prevention to evaluate the health status of the US population. Participants are recruited nationwide to guarantee sample representativeness. The NHANES protocol was approved by the Research Ethics Review Board of the National Center for Health Statistics of the United States, and written informed consent was obtained from all participants.

### 2.2. Study population

Initially, 70,190 individuals from the NHANES 2005 to 2018 cycles were enrolled in this study. We excluded participants younger than 20 years old, those with incomplete chest pain diagnostic data, and individuals missing covariate information. Notably, severe liver dysfunction can alter STB concentrations and independently affect the risk of chest pain, thereby leading to reverse causation and disease-related confounding. In accordance with previous studies, subjects with liver dysfunction (defined as aspartate aminotransferase [AST] >100 U/L, alanine aminotransferase [ALT] >100 U/L, gamma-glutamyl transferase [GGT] >100 U/L, or STB >51.3 μmol/L) and participants with self-reported liver disease were excluded.^[16]^ Finally, a total of 19,187 participants were included in the subsequent analysis (Fig. 1).

**Figure 1.**
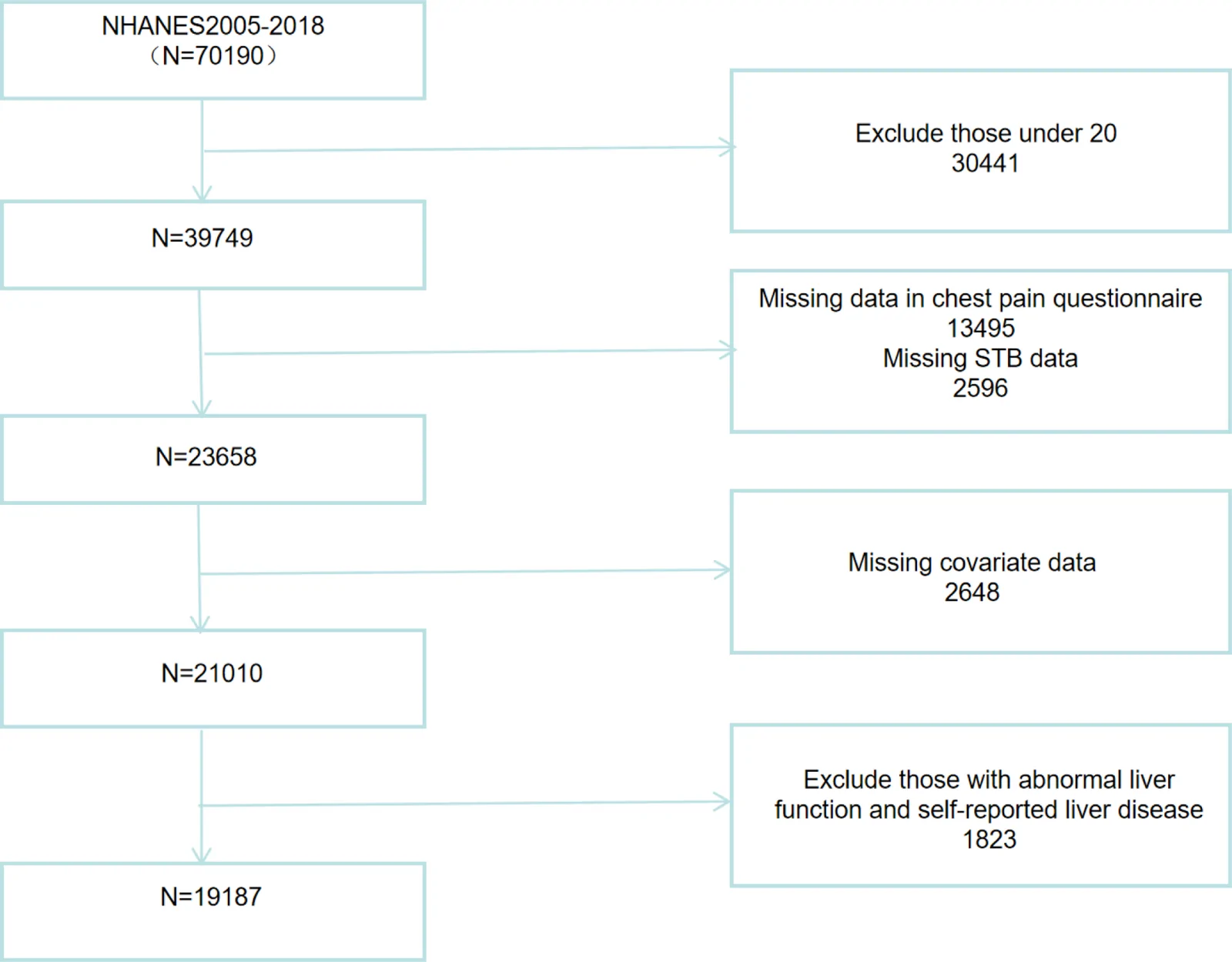
Flow diagram of the inclusion criteria and exclusion criteria. NHANES = National Health and Nutrition Examination Survey, STB = serum total bilirubin.

### 2.3. Exposure and outcome

STB concentrations were measured via the timed endpoint diazo method (Jendrassik–Grof method), and STB levels were recorded in μmol/L in the NHANES database. The primary outcome was the presence or absence of chest pain. The Standardized Rose Angina Questionnaire was used by investigators to assess chest pain symptoms of participants. Subjects who answered affirmatively to the question “Have you experienced any pain or discomfort in your chest?” were classified as having chest pain; otherwise, they were categorized into the non-chest pain group.^[17]^

### 2.4. Covariates

Covariates incorporated in this study covered age, marital status, race, smoking status, body mass index (BMI), educational attainment, diabetes mellitus, hypertension, chronic kidney disease (CKD), heart failure, CHD, myocardial infarction, angina, stroke, asthma, and depression, as well as laboratory indicators including high-density lipoprotein (HDL), total cholesterol (TC), lactate dehydrogenase (LDH), AST, ALT, and GGT. Smoking status was divided into 3 groups (current smoker, former smoker, and never smoker) based on 2 questionnaire items: “Have you smoked at least 100 cigarettes in your entire life?” and “Do you currently smoke cigarettes?” The CKD Epidemiology Collaboration formula was adopted to calculate estimated glomerular filtration rate. CKD was defined as urinary albumin-to-creatinine ratio ≥ 30 mg/g or estimated glomerular filtration rate < 60 mL/min/1.73 m^2^.^[18,19]^ Diabetes was diagnosed according to self-reported physician-diagnosed diabetes, insulin or hypoglycemic medication use, fasting blood glucose ≥ 126 mg/dL, or glycated hemoglobin ≥ 6.5%. Hypertension was defined as physician-diagnosed hypertension or average systolic blood pressure ≥ 140 mm Hg/average diastolic blood pressure > 90 mm Hg. Marital status (cohabiting/living alone), asthma, cardiovascular disease, stroke, malignancy, and depression were all determined by self-reported questionnaires. Cardiovascular disease encompassed CHD, angina, heart failure, and prior cardiovascular events (myocardial infarction).

### 2.5. Statistical analyses

Continuous variables were presented as mean ± standard deviation (SD), while categorical variables were expressed as percentages. Differences between groups were compared using the *t* test or χ^2^ test accordingly. Three sequentially adjusted logistic regression models were constructed to examine the association between STB and chest pain, with adjusted odds ratios (ORs) and 95% confidence intervals (CIs) reported. Restricted cubic spline (RCS) analysis was performed to explore the nonlinear relationship between STB and chest pain. Model 1 was unadjusted. Model 2 was adjusted for age, gender, BMI, marital status, educational level, race, and smoking status. Model 3 was further adjusted for CKD, diabetes mellitus, hypertension, heart failure, CHD, myocardial infarction, angina, stroke, asthma, depression, HDL, TC, LDH, AST, ALT, and GGT on the basis of Model 2. In addition, logistic regression and RCS analyses were repeated in 2 supplementary cohorts: 21,010 participants including individuals with liver dysfunction and self-reported liver disease, and participants without cardiovascular diseases. All statistical analyses were conducted using R software (version 4.5; R Core Team). Two-tailed *P* < .05 was considered statistically significant.

## 3. Results

### 3.1. Characteristics of the participants

A total of 19,187 adults were enrolled in the present study, and Table 1 summarizes the baseline characteristics stratified by chest pain status (chest pain vs no chest pain). Males accounted for 48.5% and females for 51.5%, with no statistically significant difference in gender distribution between the 2 groups (*P* = .086). The overall mean age was 59.83 ± 12.25 years. Compared with participants without chest pain, individuals experiencing chest pain were older and had higher BMI, LDH, and GGT levels, accompanied by lower levels of HDL, TC, STB, AST, and ALT. Subjects with chest pain exhibited higher prevalence of heart failure, angina, CHD, myocardial infarction, diabetes mellitus, hypertension, stroke, cancer, asthma, CKD, and depression. Significant intergroup differences were also observed in smoking status, marital status, and race, whereas educational attainment showed no statistically significant difference. Detailed data are presented in Table 1. Baseline characteristics of the 21,010 participants including those with liver dysfunction and self-reported liver disease are provided in Supplementary Table 1, Supplemental Digital Content 1.

**Table 1:** Baseline characteristics of participants.

	Overall	No pain	Pain	*P*
n	19,187	14,234	4953	
Age (mean [SD])	59.83 (12.25)	59.68 (12.30)	60.27 (12.08)	.004
BMI (mean [SD])	29.58 (6.70)	29.27 (6.50)	30.47 (7.16)	<.001
STB (mean [SD])	11.16 (4.88)	11.26 (4.84)	10.88 (4.96)	<.001
ALT (mean [SD])	22.64 (10.41)	22.74 (10.46)	22.36 (10.27)	.028
LDH (mean [SD])	137.23 (31.38)	136.72 (31.39)	138.69 (31.31)	<.001
AST (mean [SD])	23.97 (7.46)	24.05 (7.46)	23.74 (7.45)	.012
GGT (mean [SD])	25.02 (15.35)	24.81 (15.26)	25.61 (15.57)	.002
HDL (mean [SD])	53.73 (16.31)	54.16 (16.32)	52.49 (16.21)	<.001
TC (mean [SD])	197.48 (42.20)	198.52 (41.80)	194.47 (43.19)	<.001
Gender (%)				
Female	51.5	51.1	52.5	.086
Male	48.5	48.9	47.5	
Marital status (%)				
Living alone	36.9	35.5	40.9	<.001
Cohabiting	63.1	64.5	59.1	
Education (%)				
Below high school	26	25.9	26.3	.556
High school or above	74	74.1	73.7	
Race (%)				
Other	32.8	34.7	27.5	<.001
Non-Hispanic White	45.9	44.6	49.7	
Non-Hispanic Black	21.2	20.7	22.8	
Smoking status (%)				
Current smoker	17.6	16.1	21.8	<.001
Former smoker	29.9	28.9	32.7	
Never smoker	52.5	55	45.5	
CHD (%)				
No	94.1	97	85.7	<.001
Yes	5.9	3	14.3	
Angina (%)				
No	96.5	98.9	89.7	<.001
Yes	3.5	1.1	10.3	
Heart failure (%)				
No	95.7	97.6	90	<.001
Yes	4.3	2.4	10	
Myocardial infarction (%)				
No	94.2	97.5	84.6	<.001
Yes	5.8	2.5	15.4	
Hypertension (%)				
No	45.7	48.7	37.3	<.001
Yes	54.3	51.3	62.7	
Stroke (%)				
No	95.1	96.3	91.6	<.001
Yes	4.9	3.7	8.4	
Diabetes (%)				
No	76.7	77.9	73.2	<.001
Yes	23.3	22.1	26.8	
CKD (%)				
No	85.4	86.4	82.6	<.001
Yes	14.6	13.6	17.4	
Cancer (%)				
No	86.8	87.6	84.6	<.001
Yes	13.2	12.4	15.4	
Asthma (%)				
No	87.1	89.7	79.6	<.001
Yes	12.9	10.3	20.4	
Depression (%)				
No	91.7	94.6	83.5	<.001
Yes	8.3	5.4	16.5	

ALT = alanine aminotransferase, AST = aspartate aminotransferase, BMI = body mass index, CHD = coronary heart disease, CKD = chronic kidney disease, GGT = gamma-glutamyl transferase, HDL = high-density lipoprotein, LDH = lactate dehydrogenase, SD = standard deviation, STB = serum total bilirubin, TC = total cholesterol.

### 3.2. Association between STB and chest pain

The associations between STB and chest pain risk are summarized in Table 2. In Model 1 without covariate adjustment, log(STB) was negatively correlated with chest pain (OR = 0.87, 95% CI: 0.82–0.91, *P* < .001). After adjusting for demographic variables in Model 2 (OR = 0.90, 95% CI: 0.86–0.95, *P* < .001) and full covariate adjustment in Model 3 (OR = 0.91, 95% CI: 0.85–0.96, *P* < .001), the association between STB and chest pain remained statistically significant. In Model 3, compared with quartile 1 (Q1), the ORs and corresponding 95% CIs for the association between quartile 2 (Q2), quartile 3 (Q3), and quartile 4 (Q4) and chest pain were 0.92 (0.82–1.03), 0.87 (0.79–0.96), and 0.86 (0.78–0.95), respectively.

**Table 2:** Association between STB and chest pain.

Characteristic	Model 1	Model 2	Model 3
	OR (95% CI), *P* value	OR (95% CI), *P* value	OR (95% CI), *P* value
log_2_(STB) (continuous)	0.87 (0.82–0.91), <.001	0.90 (0.86–0.95), <.001	0.91 (0.85–0.96), <.001
STB Q1	Reference	Reference	Reference
STB Q2	0.91 (0.82–1.01), .072	0.91 (0.82–1.02), .099	0.92 (0.82–1.03), .136
STB Q3	0.83 (0.76–0.91), <.001	0.84 (0.78–0.93), <.001	0.87 (0.79–0.96), .006
STB Q4	0.79 (0.72–0.86), <.001	0.85 (0.77–0.93), <.001	0.86 (0.78–0.95), .008
*P* for trend	<.001	.001	.014

Model 1: unadjusted.

Model 2: adjusted for age, BMI, sex, marital status, educational level, race, and smoking status.

Model 3: adjusted for all covariates included in Model 2 plus CKD, hypertension, diabetes mellitus, CHD, angina pectoris, heart failure, myocardial infarction, stroke, asthma, cancer, depression, total cholesterol, HDL, LDH, AST, ALT, and GGT.

ALT = alanine aminotransferase, AST = aspartate aminotransferase, BMI = body mass index, CHD = coronary heart disease, CI = confidence interval, CKD = chronic kidney disease, GGT = gamma-glutamyl transferase, HDL = high-density lipoprotein, LDH = lactate dehydrogenase, OR = odds ratio, STB = serum total bilirubin.

### 3.3. RCS curve for the association between STB and chest pain

The RCS curve demonstrated a nonlinear association between STB and the risk of chest pain (*P* for nonlinearity = .008; Fig. 2). A threshold value of 14.42 μmol/L was identified. When STB was below 14.42 μmol/L, elevated STB was associated with a reduced risk of chest pain (OR = 0.91, 95% CI: 0.86–0.96). No significant correlation was observed between STB and chest pain risk when STB was ≥14.42 μmol/L (OR = 1.21, 95% CI: 0.90–1.62).

**Figure 2.**
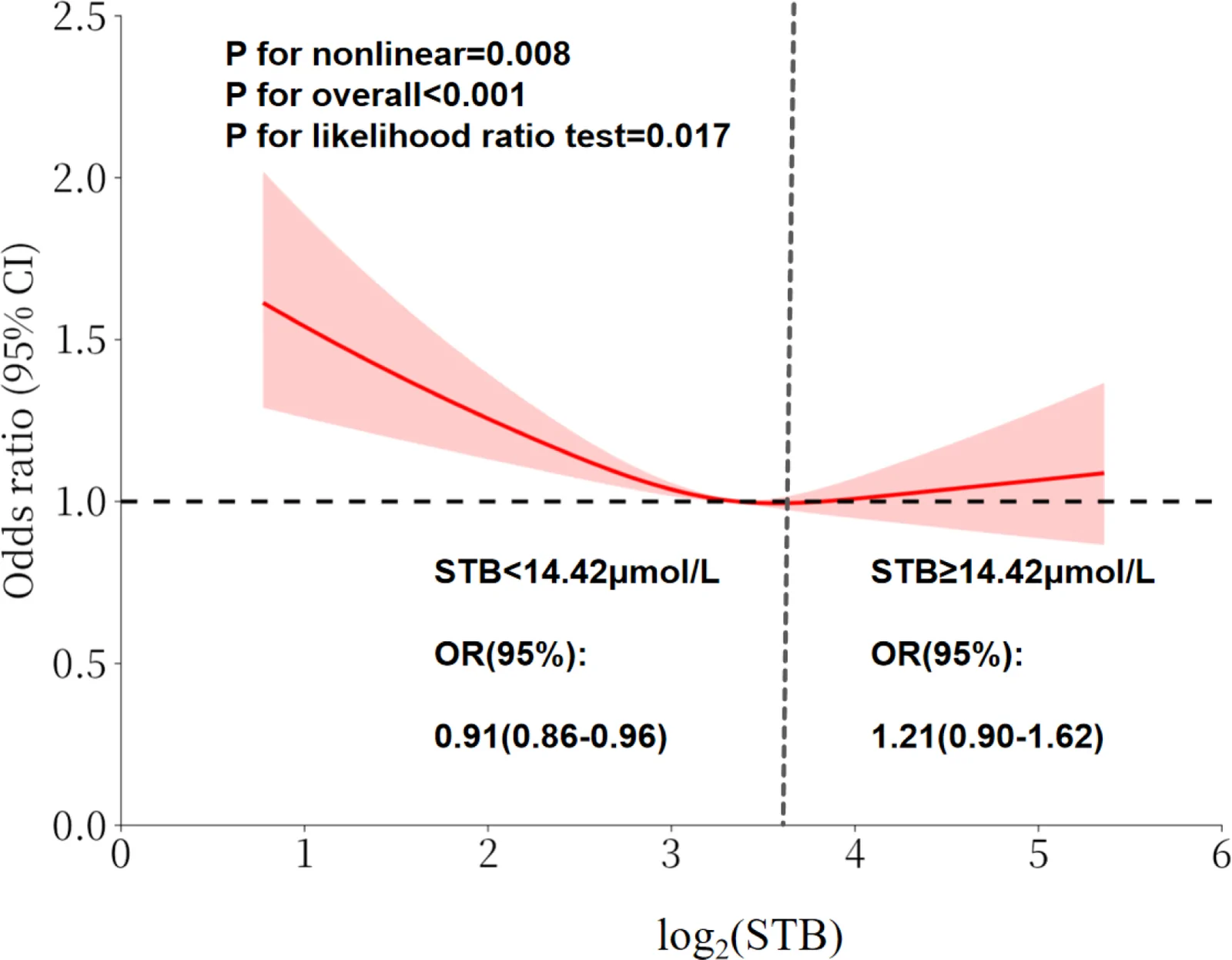
RCS curve depicting the association between STB and chest pain risk. Smooth curve fitting plot for the dose–response relationship between STB and chest pain; adjusted for age, gender, BMI, marital status, educational level, ethnicity, smoking status, CKD, hypertension, diabetes mellitus, CHD, angina pectoris, heart failure, myocardial infarction, stroke, asthma, cancer, depression, total cholesterol, HDL, LDH, AST, ALT, and GGT. ALT = alanine aminotransferase, AST = aspartate aminotransferase, BMI = body mass index, CHD = coronary heart disease, CI = confidence interval, CKD = chronic kidney disease, GGT = gamma-glutamyl transferase, HDL = high-density lipoprotein, LDH = lactate dehydrogenase, OR = odds ratio, RCS = restricted cubic spline, STB = serum total bilirubin.

### 3.4. Subgroup analyses

The results of subgroup interaction analyses are presented in Figure 3. The significant association between STB and chest pain persisted in females, obese individuals, participants without hypertension, diabetes, stroke, CKD, malignancy, asthma, or depression, as well as current and former smokers. Notably, a significant interaction was detected between normal/overweight and obesity subgroups (*P* for interaction = .043). STB was significantly negatively correlated with chest pain in the obese subgroup, whereas no statistically significant association was found in the normal-weight and overweight subgroups.

**Figure 3.**
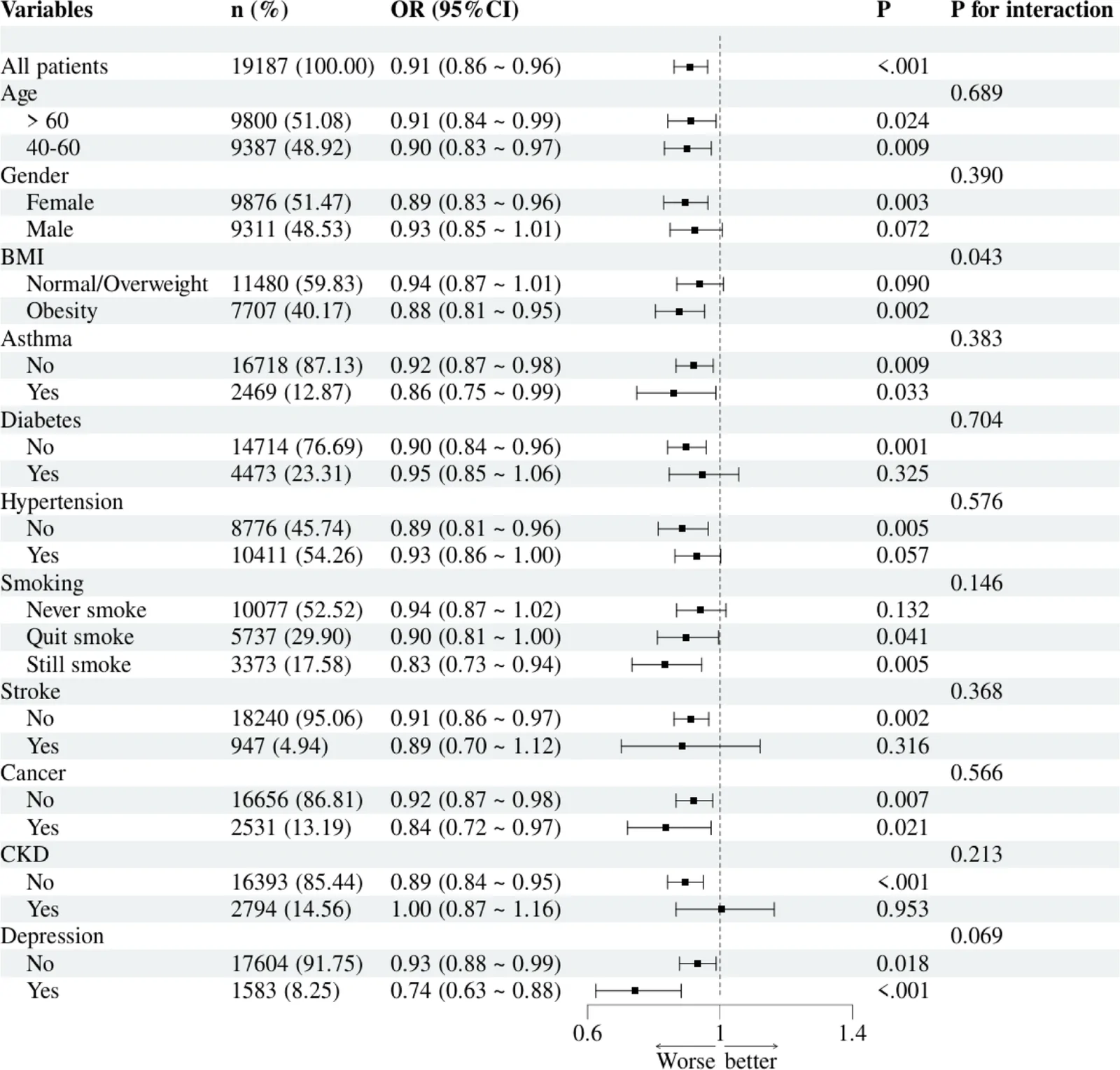
Forest plots depicting subgroup analysis of STB and chest‑pain risk. Forest plots were used for subgroup analysis; normal weight and overweight were defined as a BMI <30; obesity was defined as a BMI ≥30. BMI = body mass index, CI = confidence interval, CKD = chronic kidney disease, OR = odds ratio, STB = serum total bilirubin.

### 3.5. Sensitivity analysis

We conducted 3 supplementary analyses using logistic regression and RCS curves. The first analysis comprised 21,010 participants, including subjects with liver dysfunction and self-reported liver disease (Supplementary Tables 1 and 2, Supplemental Digital Content 1; Supplementary Fig. 1, Supplemental Digital Content 3). The second analysis included 16,952 participants after excluding individuals with cardiovascular diseases (Supplementary Table 3, Supplemental Digital Content 4; Supplementary Fig. 2, Supplemental Digital Content 5). The third analysis evaluated the association between STB and chest pain risk by incorporating daily alcohol intake into the model (Supplementary Tables 4 and 5, Supplemental Digital Content 6; Supplementary Figs. 3 and 4, Supplemental Digital Content 8). Findings from all 3 supplementary analyses were consistent with those from the primary analysis, further supporting our conclusions.

## 4. Discussion

For the first time, we explored the association between STB and chest pain symptoms based on a large sample size and identified an inverse dose–response relationship between chest pain and STB concentrations in our study population. Threshold effect analysis further revealed that STB levels below 14.42 μmol/L were linked to a lower risk of chest pain, whereas no significant association was observed when STB was ≥14.42 μmol/L. These findings may carry important implications for risk assessment of chest pain.

Existing studies have verified a potential link between STB and diseases manifesting with chest pain. In a prospective cohort of 7222 participants, Kunutsor et al found that each SD increment in STB was associated with an 11% reduction in cardiovascular disease risk. Their meta-analysis further demonstrated that per SD rise in STB reduced the overall risk of cardiovascular disease by 7% and the risk of CHD by 5%.^[20]^ Kang et al reported that patients with hyperbilirubinemia (>1.2 mg/dL) had a markedly lower prevalence of atherosclerotic stenosis >50% and calcified plaques compared with those in the low-bilirubin group (≤1.2 mg/dL).^[21]^ Gullu et al confirmed that higher physiological STB concentrations were associated with superior coronary flow reserve in healthy young adults, indicating a potential protective effect of STB against coronary microvascular injury and atherosclerosis.^[22]^ Another study revealed that elevated STB was associated with favorable collateral vessel development in patients with chronic total coronary occlusion.^[23]^ A cross-sectional study observed significantly lower STB concentrations among patients with atrial fibrillation relative to controls, and the anti-inflammatory and antioxidant properties of bilirubin were proposed as the underlying mechanism.^[24]^ Another investigation suggested that direct bilirubin might serve as a potential predictor for pulmonary embolism following hip surgery.^[25]^ A case–control study indicated that individuals with STB <8.6 μmol/L exhibited a 73% higher risk of pulmonary embolism relative to controls.^[26]^ Oskay et al discovered reduced STB levels in patients with herpes zoster versus healthy controls.^[15]^ In contrast, another study showed that subjects with STB ≥9.2 μmol/L had a 69.8% lower risk of developing postherpetic neuralgia.^[27]^

Our findings are largely consistent with previous literature. Logistic regression and subgroup analyses were performed to explore the relationship between STB and chest pain in the current study. Our results indicated that elevated STB was inversely correlated with the prevalence of chest pain. Subgroup analyses identified a significant difference in the STB–chest pain association between obese individuals and those with normal weight or overweight; the correlation was stronger within the obese population, which may suggest a protective effect of STB against obesity and obesity-related metabolic disorders.^[28,29]^ Nevertheless, owing to the multiple subgroup analyses conducted and the lack of definitive mechanistic explanations, this observation requires further validation.

Several plausible mechanisms may explain the association between STB and chest pain. First, given the anti-inflammatory and antioxidant properties of STB, higher STB concentrations may improve coronary endothelial function by eliminating reactive oxygen species and enhancing nitric oxide bioavailability.^[22,30–32]^ Furthermore, McArdle et al demonstrated that the UGT1A1*28 genotype, a key genetic variant governing bilirubin metabolism, was correlated with brachial artery diameter and cold pressor reactivity, implying that STB exerts early regulatory effects on vascular structure and vascular responsiveness.^[33]^ Second, prior research has confirmed that oxidative stress and inflammatory markers (such as albumin-to-red cell distribution width ratio and neutrophil-to-albumin ratio) are related to the incidence and prognosis of chest pain, and systemic oxidative stress and inflammatory burden can promote the development of chest pain. Third, under oxidative stress, activated platelets aggregate to trigger thrombosis while exacerbating inflammation and endothelial dysfunction; the antioxidant capacity of STB may contribute to regulating platelet function in vivo.^[34–36]^ In addition, as a biomarker of inflammation and oxidative stress, STB has been found to correlate with psychiatric disorders,^[37,38]^ pulmonary embolism,^[26,39]^ and herpes zoster,^[15,27]^ suggesting that STB may be involved in multiple etiologies responsible for chest pain. However, precise biological mechanisms remain to be clarified through additional experimental and longitudinal studies.

Notably, albumin also participates in the pathogenesis of chest pain and possesses long-term prognostic predictive value.^[40–42]^ Sulfhydryl groups within albumin scavenge reactive oxygen species, inhibit lipid peroxidation, and suppress the secretion of pro-inflammatory factors including tumor necrosis factor-α and interleukin-6, thereby protecting blood vessels and stabilizing atherosclerotic plaques.^[43]^ Hypoalbuminemia-induced malnutrition exacerbates systemic frailty and consequently increases the risk of chest pain,^[44,45]^ and albumin also carries independent prognostic value.^[40]^ STB reflects systemic oxidative stress status, while albumin reflects systemic anti-inflammatory and antioxidant capacity and nutritional reserves. Multiple studies have reported associations between plasma bilirubin levels and all-cause mortality,^[46,47]^ implying that bilirubin and albumin may share overlapping functional pathways.

The RCS curves revealed a nonlinear relationship between STB and chest pain, and segmented regression identified a distinct inflection point at 14.42 μmol/L. STB was negatively associated with chest pain below this threshold, whereas no significant correlation was observed above 14.42 μmol/L. Sensitivity analyses yielded results consistent with the primary analyses. This nonlinear pattern has also been observed in the associations between STB and other chronic diseases.^[48–50]^ A possible explanation is that once STB exceeds this threshold, its antioxidant property shifts toward cytotoxicity.^[51]^

The present study had a sufficient sample size, and multiple potential confounding factors were adjusted for in the statistical analyses. The consistent results across subgroup analyses and sensitivity analyses support the robustness of our conclusions. Nevertheless, several limitations should be acknowledged. First, the chest pain questionnaire adopted by NHANES cannot distinguish the type and nature of chest pain, making it impossible to clarify the associations between STB and different subtypes of chest pain. Although the overall sample size was large, participants with missing data were excluded during analysis. Furthermore, covariates such as alcohol consumption and physical activity were excluded from the regression models due to high missing rates, which might introduce selection bias. Second, substantial missing data were present in the Rose questionnaire chest pain module within the NHANES database, so chest pain was defined by only a single item in this study. Although this definition has been validated in other large cohort studies, recall bias and misunderstanding of questionnaire items among participants may lead to misclassification of the outcome. Third, STB was not stratified in our analysis; we failed to explore the separate relationships of direct bilirubin and indirect bilirubin with chest pain. Targeted research focusing on these 2 fractions of bilirubin can be carried out in future work. Fourth, Gilbert syndrome, also known as benign hyperbilirubinemia, may serve as a critical confounder in this study. Patients with Gilbert syndrome may benefit from mild hyperbilirubinemia, including reduced risks of cardiovascular diseases and other metabolic disorders.^[52,53]^ Due to the limited data availability of the public database, we could not identify or adjust for individuals with Gilbert syndrome, and omission of this confounder may distort the effect estimate for the association between STB and chest pain. Finally, although our results demonstrated that higher bilirubin levels were correlated with a lower risk of chest pain, it must be emphasized that causal inference cannot be drawn from observational data. As a cross-sectional study, this research only explored associations rather than causal relationships. It remains unclear whether abnormal bilirubin levels trigger chest pain or whether chest pain-related lesions alter bilirubin metabolism. Rigorous experimental studies and longitudinal cohorts are required to determine causality in the future.

## 5. Conclusion

In our study population, elevated STB was associated with a lower risk of chest pain, accompanied by a significant nonlinear relationship and threshold effect at an STB concentration of 14.42 μmol/L. Below this cutoff value, bilirubin was inversely correlated with chest pain risk, indicating that bilirubin may have potential applications in chest pain risk stratification. Nevertheless, owing to the inherent limitations of the chest pain questionnaire in the NHANES database, chest pain was not classified as cardiac or noncardiac, intermittent or persistent. Hence, our findings should be interpreted cautiously. Comprehensive investigations into the relationship between STB and distinct chest pain subtypes are warranted in future research. Additionally, further prospective studies are needed to establish the causal relationship between bilirubin and chest pain.

## Author contributions

**Conceptualization:** Zhenjie Fan, Zhiyong Wang, Yiwen He, Yan Cheng, Jiaxue Tang, Qian Min.

**Data curation:** Zhenjie Fan, Zhiyong Wang, Jun You, Yiwen He, Yan Cheng, Jiaxue Tang.

**Formal analysis:** Zhenjie Fan, Zhiyong Wang, Jun You, Yiwen He, Yan Cheng, Jiaxue Tang.

**Investigation:** Zhenjie Fan, Zhiyong Wang, Jun You, Yiwen He, Yan Cheng, Jiaxue Tang, Qian Min.

**Methodology:** Zhenjie Fan, Zhiyong Wang, Jun You, Yiwen He.

**Project administration:** Zhenjie Fan.

**Resources:** Zhenjie Fan, Zhiyong Wang, Yiwen He.

**Software:** Zhenjie Fan.

**Supervision:** Qian Min.

**Validation:** Zhenjie Fan, Qian Min.

**Visualization:** Zhenjie Fan, Jun You.

**Writing – original draft:** Zhenjie Fan, Zhiyong Wang, Jun You, Yiwen He, Yan Cheng, Jiaxue Tang, Qian Min.

**Writing – review & editing:** Zhenjie Fan, Zhiyong Wang, Jun You, Yiwen He, Yan Cheng, Jiaxue Tang, Qian Min.


















