# Supplementary material for: Association between chest pain and serum total bilirubin levels in adults: Cross-sectional analysis of NHANES 2005 to 2018
Source: Medicine (Baltimore). 2026 Oct 23;105(43):e50772. doi: 10.1097/MD.0000000000050772 (PMC13706258; doi:10.1097/MD.0000000000050772)
Supplement: Supplementary file 1 [file medi-105-e50772-s001.docx]

**Supplementary Table 1**:Baseline characteristics table for analysis incorporating participants with abnormal liver function

|  | Overall | No pain | pain | p |
| --- | --- | --- | --- | --- |
| n | 21010 | 15366 | 5644 |  |
| **Age (mean (SD))** | 59.69 (12.14) | 59.58 (12.22) | 59.98 (11.93) | 0.036 |
| **BMI (mean (SD))** | 29.63 (6.73) | 29.31 (6.51) | 30.49 (7.21) | <0.001 |
| **STB (mean (SD))** | 11.25 (5.14) | 11.35 (5.14) | 10.97 (5.15) | <0.001 |
| **ALT (mean (SD))** | 24.61 (19.61) | 24.51 (17.23) | 24.88 (24.96) | 0.223 |
| **LDH (mean (SD))** | 138.12 (33.16) | 137.44 (33.17) | 139.97 (33.07) | <0.001 |
| **AST (mean (SD))** | 25.73 (15.72) | 25.61 (13.91) | 26.04 (19.84) | 0.082 |
| **GGT (mean (SD))** | 32.04 (46.19) | 31.03 (44.00) | 34.79 (51.59) | <0.001 |
| **HDL (mean (SD))** | 53.69 (16.65) | 54.12 (16.61) | 52.49 (16.70) | <0.001 |
| **TC (mean (SD))** | 197.37 (42.76) | 198.43 (42.33) | 194.50 (43.79) | <0.001 |
| **Gender (%)** |  |  |  |  |
| Female | 50.9 | 50.6 | 51.8 | 0.129 |
| Male | 49.1 | 49.4 | 48.2 |  |
| **Marital status (%)** |  |  |  |  |
| No | 37.4 | 35.8 | 41.6 | <0.001 |
| Yes | 62.6 | 64.2 | 58.4 |  |
| **Education (%)** |  |  |  |  |
| Below high school | 26.2 | 26.1 | 26.6 | 0.474 |
| High School or above | 73.8 | 73.9 | 73.4 |  |
| **Race (%)** |  |  |  |  |
| Other | 33.5 | 35.2 | 28.7 | <0.001 |
| Non-Hispanic White | 45.6 | 44.3 | 49.1 |  |
| Non-Hispanic Black | 21 | 20.5 | 22.2 |  |
| **Smoking status (%)** |  |  |  |  |
| Still smoke | 18.4 | 16.8 | 22.9 | <0.001 |
| Quit smoke | 30.2 | 29.2 | 32.7 |  |
| Never smoke | 51.4 | 54 | 44.4 |  |
| **CHD (%)** |  |  |  |  |
| No | 94 | 97.1 | 85.8 | <0.001 |
| Yes | 6 | 2.9 | 14.2 |  |
| **Angina (%)** |  |  |  |  |
| No | 96.4 | 98.9 | 89.3 | <0.001 |
| Yes | 3.6 | 1.1 | 10.7 |  |
| **Heart failure (%)** |  |  |  |  |
| No | 95.4 | 97.6 | 89.6 | <0.001 |
| Yes | 4.6 | 2.4 | 10.4 |  |
| **Myocardium infarction(%)** |  |  |  |  |
| No | 94 | 97.4 | 84.5 | <0.001 |
| Yes | 6 | 2.6 | 15.5 |  |
| **Hypertension (%)** |  |  |  |  |
| No | 44.9 | 48 | 36.6 | <0.001 |
| Yes | 55.1 | 52 | 63.4 |  |
| Stroke (%) |  |  |  |  |
| No | 94.9 | 96.1 | 91.4 | <0.001 |
| Yes | 5.1 | 3.9 | 8.6 |  |
| **Diabetes (%)** |  |  |  |  |
| No | 75.8 | 77.2 | 71.9 | <0.001 |
| Yes | 24.2 | 22.8 | 28.1 |  |
| **CKD (%)** |  |  |  |  |
| No | 85.4 | 86.3 | 83 | <0.001 |
| Yes | 14.6 | 13.7 | 17 |  |
| **Carcer (%)** |  |  |  |  |
| No | 86.9 | 87.6 | 84.7 | <0.001 |
| Yes | 13.1 | 12.4 | 15.3 |  |
| **Asthma (%)** |  |  |  |  |
| No | 86.7 | 89.6 | 78.7 | <0.001 |
| Yes | 13.3 | 10.4 | 21.3 |  |
| **Depression (%)** |  |  |  |  |
| No | 91 | 94.2 | 82.2 | <0.001 |
| Yes | 9 | 5.8 | 17.8 |  |
| **Liver disease (%)** |  |  |  |  |
| No | 94.8 | 96 | 91.5 | <0.001 |
| Yes | 5.2 | 4 | 8.5 |  |

STB：serum total bilirubin;AST：Aspartate Aminotransferase;ALT：Alanine Aminotransferase;GGT：Gamma-Glutamyl Transferase;LDH：Lactate Dehydrogenase;HDL：High-Density Lipoprotein;TC：Total Cholesterol;CKD：Chronic Kidney Disease;CHD：Coronary Heart Disease
