# Supplementary material for: Association between chest pain and serum total bilirubin levels in adults: Cross-sectional analysis of NHANES 2005 to 2018
Source: Medicine (Baltimore). 2026 Oct 23;105(43):e50772. doi: 10.1097/MD.0000000000050772 (PMC13706258; doi:10.1097/MD.0000000000050772)
Supplement: Supplementary file 2 [file medi-105-e50772-s002.docx]

**Supplementary Table 2**: Association between STB and chest pain when incorporating participants with abnormal liver function

| Characteristic | Model 1 | Model 2 | Model 3 |
| --- | --- | --- | --- |
|  | OR (95%CI)P value | OR (95%CI)P value | OR (95%CI)P value |
| log_2_(STB)(continuous) | 0.86 (0.83 0.90) <0.001 | 0.90 (0.86 0.94) <0.001 | 0.90 (0.85 0.95) <0.001 |
| STB Q1 | reference | reference | reference |
| STB Q2 | 0.87 （0.79 0.96) 0.007 | 0.88 (0.80 0.97) 0.013 | 0.89 (0.80 0.99) 0.031 |
| STB Q3 | 0.81 （0.75 0.88) <0.001 | 0.83（0.76 0.90) <0.001 | 0.88（0.78 0.94) <0.001 |
| STB Q4 | 0.78 （0.72 0.85) <0.001 | 0.84 (0.77 0.91) <0.001 | 0.91 (0.78 0.94) 0.002 |
| P for Trend | <0.001 | 0.001 | 0.016 |

Model 1: unadjusted.

Model 2: adjusted for age, BMI, sex, marital status, educational level, race and smoking status.

Model 3: adjusted for all covariates in Model 2, additionally adjusted for CKD, hypertension, diabetes mellitus, CHD, angina pectoris, heart failure, myocardial infarction, stroke, asthma, cancer, depression, total cholesterol, HDL, LDH, AST, ALT, GGT and liver disease.
