# Supplementary material for: Association between chest pain and serum total bilirubin levels in adults: Cross-sectional analysis of NHANES 2005 to 2018
Source: Medicine (Baltimore). 2026 Oct 23;105(43):e50772. doi: 10.1097/MD.0000000000050772 (PMC13706258; doi:10.1097/MD.0000000000050772)
Supplement: Supplementary file 3 [file medi-105-e50772-s004.docx]

| **All-cause mortality** | Model 1 | Model 2 | Model 3 |
| --- | --- | --- | --- |
|  | OR (95%CI)P value | OR (95%CI)P value | OR (95%CI)P value |
| **log（STB） (continuous)** | 0.83 (0.78 0.87) <0.001 | 0.89 (0.84 0.94) <0.001 | 0.90 (0.85 0.96) <0.001 |
| **STB Q1** | reference | reference | reference |
| **STB Q2** | 0.88 (0.78 0.99) 0.031 | 0.89 (0.79 1.00) 0.056 | 0.90 (0.79 1.01) 0.077 |
| **STB Q3** | 0.80（0.73 0.88) <0.001 | 0.84（0.76 0.93) 0.001 | 0.87（0.78 0.96) 0.008 |
| **STB Q4** | 0.73（0.66 0.81) <0.001 | 0.85 (0.74 0.92) <0.001 | 0.86 (0.77 0.96) 0.009 |
| **P for Trend** | <0.001 | 0.011 | 0.02 |

**Supplementary Table 3**: Association between STB and chest pain after excluding participants with cardiovascular diseases

Model 1: unadjusted.

Model 2: adjusted for age, BMI, sex, marital status, educational level, race and smoking status.

Model 3: adjusted for all covariates in Model 2 plus CKD, hypertension, diabetes mellitus, stroke, asthma, cancer, depression, total cholesterol, HDL, LDH, AST, ALT and GGT.
