# Supplementary material for: Association between chest pain and serum total bilirubin levels in adults: Cross-sectional analysis of NHANES 2005 to 2018
Source: Medicine (Baltimore). 2026 Oct 23;105(43):e50772. doi: 10.1097/MD.0000000000050772 (PMC13706258; doi:10.1097/MD.0000000000050772)
Supplement: Supplementary file 4 [file medi-105-e50772-s006.docx]

**Supplementary Table 4:**Baseline characteristics table excluding participants missing alcohol intake data

|  | Overall | No pain | pain | p |
| --- | --- | --- | --- | --- |
| n | 15583 | 11551 | 4032 |  |
| Age (mean (SD)) | 59.64 (12.21) | 59.47 (12.26) | 60.12 (12.05) | 0.004 |
| BMI (mean (SD)) | 29.69 (6.77) | 9.35 (6.57) | 30.64 (7.25) | <0.001 |
| Alcohol intake(mean (SD)) | 8.40 (23.11) | 8.63 (23.14) | 7.75 (23.00) | 0.039 |
| STB (mean (SD)) | 10.81 (4.79) | 10.91 (4.74) | 10.51 (4.90) | <0.001 |
| ALT (mean (SD)) | 22.56 (10.44) | 22.67 (10.51) | 22.25 (10.25) | 0.028 |
| LDH (mean (SD)) | 137.29 (32.03) | 136.82 (32.05) | 138.64 (31.95) | 0.002 |
| AST (mean (SD)) | 23.79 (7.46) | 23.88 (7.44) | 23.53 (7.50) | 0.009 |
| GGT (mean (SD)) | 24.78 (15.26) | 24.56 (15.18) | 25.41 (15.47) | 0.002 |
| HDL (mean (SD)) | 53.92 (16.42) | 54.39 (16.44) | 52.57 (16.28) | <0.001 |
| TC (mean (SD)) | 196.92 (42.32) | 198.05 (42.07) | 193.70 (42.89) | <0.001 |
| Gender (%) |  |  |  |  |
| Female | 51.3 | 51 | 52.3 | 0.169 |
| Male | 48.7 | 49 | 47.7 |  |
| Marital status (%) |  |  |  |  |
| Living alone | 37 | 35.4 | 41.5 | <0.001 |
| Cohabiting | 63 | 64.6 | 58.5 |  |
| Education (%) |  |  |  |  |
| Below high school | 24.7 | 24.6 | 24.8 | 0.794 |
| High School or above | 75.3 | 75.4 | 75.2 |  |
| Race (%) |  |  |  |  |
| Other | 32.8 | 34.5 | 27.9 | <0.001 |
| Non-Hispanic White | 45.5 | 44.5 | 48.5 |  |
| Non-Hispanic Black | 21.7 | 21 | 23.6 |  |
| Smoking status (%) |  |  |  |  |
| Still smoke | 52.8 | 55.3 | 45.8 | <0.001 |
| Quit smoke | 29.8 | 28.9 | 32.3 |  |
| Never smoke | 17.4 | 15.9 | 21.9 |  |
| CHD (%) |  |  |  |  |
| No | 94 | 97 | 85.7 | <0.001 |
| Yes | 6 | 3 | 14.3 |  |
| Angina (%) |  |  |  |  |
| No | 96.6 | 98.9 | 89.7 | <0.001 |
| Yes | 3.4 | 1.1 | 10.3 |  |
| Heart failure (%) |  |  |  |  |
| No | 95.7 | 97.7 | 89.9 | <0.001 |
| Yes | 4.3 | 2.3 | 10.1 |  |
| Myocardium infarction(%) |  |  |  |  |
| No | 94.2 | 97.5 | 84.5 | <0.001 |
| Yes | 5.8 | 2.5 | 15.5 |  |
| Hypertension (%) |  |  |  |  |
| No | 45.8 | 48.7 | 37.4 | <0.001 |
| Yes | 54.2 | 51.3 | 62.6 |  |
| Stroke (%) |  |  |  |  |
| No | 95.1 | 96.4 | 91.5 | <0.001 |
| Yes | 4.9 | 3.6 | 8.5 |  |
| Diabetes (%) |  |  |  |  |
| No | 76.8 | 77.9 | 73.5 | <0.001 |
| Yes | 23.2 | 22.1 | 26.5 |  |
| CKD (%) |  |  |  |  |
| No | 85.6 | 86.6 | 82.8 | <0.001 |
| Yes | 14.4 | 13.4 | 17.2 |  |
| Carcer (%) |  |  |  |  |
| No | 86.8 | 87.5 | 84.8 | <0.001 |
| Yes | 13.2 | 12.5 | 15.2 |  |
| Asthma (%) |  |  |  |  |
| No | 86.9 | 89.6 | 79.1 | <0.001 |
| Yes | 13.1 | 10.4 | 20.9 |  |
| Depression (%) |  |  |  |  |
| No | 91.9 | 94.8 | 83.4 | <0.001 |
| Yes | 8.1 | 5.2 | 16.6 |  |

STB：serum total bilirubin;AST：Aspartate Aminotransferase;ALT：Alanine Aminotransferase;GGT：Gamma-Glutamyl Transferase;LDH：Lactate Dehydrogenase;HDL：High-Density Lipoprotein;TC：Total Cholesterol;CKD：Chronic Kidney Disease;CHD：Coronary Heart Disease
