# Supplementary material for: Association between chest pain and serum total bilirubin levels in adults: Cross-sectional analysis of NHANES 2005 to 2018
Source: Medicine (Baltimore). 2026 Oct 23;105(43):e50772. doi: 10.1097/MD.0000000000050772 (PMC13706258; doi:10.1097/MD.0000000000050772)
Supplement: Supplementary file 5 [file medi-105-e50772-s007.docx]

**Supplementary Table 5：**Association between STB and Chest Pain excluding participants missing alcohol intake data

| All-cause mortality | Model 1 | Model 2 | Model 3 |
| --- | --- | --- | --- |
|  | OR (95%CI)P value | OR (95%CI)P value | OR (95%CI)P value |
| log（STB） (continuous) | 0.86 (0.81 0.90) <0.001 | 0.90 (0.85 0.95) <0.001 | 0.90 (0.85 0.96) <0.001 |
| STB Q1 | reference | reference | reference |
| STB Q2 | 0.86 (0.77 0.97) 0.011 | 0.87 (0.77 0.98) 0.019 | 0.84 (0.74 0.74) 0.005 |
| STB Q3 | 0.78（0.76 0.91) <0.001 | 0.78（0.71 0.90) <0.001 | 0.80（0.79 0.96) <0.001 |
| STB Q4 | 0.71（0.63 0.80) <0.001 | 0.78 (0.69 0.88) <0.001 | 0.77 (0.68 0.88) <0.001 |
| P for Trend | <0.001 | 0.001 | 0.004 |

Model 1: unadjusted.

Model 2: adjusted for age, BMI, sex, marital status, educational level, race, smoking status and alcohol intake.

Model 3: adjusted for all covariates included in Model 2 plus CKD, hypertension, diabetes mellitus, CHD, angina pectoris, heart failure, myocardial infarction, stroke, asthma, cancer, depression, total cholesterol, HDL, LDH, AST, ALT and GGT.
