# Supplementary material for: Association between chest pain and serum total bilirubin levels in adults: Cross-sectional analysis of NHANES 2005 to 2018
Source: Medicine (Baltimore). 2026 Oct 23;105(43):e50772. doi: 10.1097/MD.0000000000050772 (PMC13706258; doi:10.1097/MD.0000000000050772)
Supplement: Supplementary file 6 [file medi-105-e50772-s009.docx]

 Association Between Chest Pain and serum total bilirubin Levels in Adults: Cross‑Sectional Analysis of NHANES 2005–2018

**Zhenjie Fan MM^1,2^, Zhiyong Wang MM^1,2^,Jun You MM^1,2^, Yiwen He MM^1,2^, Yan Cheng MM^1,2^,JiaXue Tang MM^1,2^ ,Min Qian MM^1,2*^**

1:The First College of Clinical Medical Science, China Three Gorges University, Yichang, China

2：Department of Emergency, Yichang Central People’s Hospital, Yichang, China

**^*:^**Corresponding author, Email : [qianmin@ctgu.edu.cn](mailto:qianmin@ctgu.edu.cn.cheng)；ORCID：0009-0005-9056-3177;

Email

Zhenjie Fan:1375075592@qq.com

Zhiyon Wang:202310510021092@ctgu.edu.cn;

Jun You:junyouquq@foxmail.com

Yiwen He:603284257@qq.com

JiaXue Tang:nevatang@163.com;

Yan.Cheng:chengyan198682@163.com;Master of Medicine

| STB | serum total bilirubin |
| --- | --- |
| AST | Aspartate Aminotransferase |
| ALT | Alanine aminotransferasee |
| GGT | Gamma-Glutamyl Transferase |
| LDH | Lactate Dehydrogenase |
| HDL | High-Density Lipoprotein |
| TC | Total Cholesterol |
| CKD | Chronic Kidney Disease |
| CHD | Coronary Heart Disease |
| NHANES | National Health and Nutrition Examination Survey |
| RCS | Restricted Cubic Spline |
| CDC | Centers for Disease Control and Prevention |

**Abstract**

The association between serum total bilirubin (STB) and chest pain remains unclear. This study aimed to explore the potential relationship between STB and the prevalence of chest pain.

Data from the National Health and Nutrition Examination Survey (NHANES) 2005–2018 were analyzed to evaluate the association between STB levels and chest pain. Logistic regression (odds ratio [OR] with 95% confidence interval [CI]), restricted cubic spline (RCS) curves and stratified subgroup analyses were performed, and a total of 19,187 participants were enrolled. STB was negatively correlated with chest pain; each unit increment in log₂(STB) corresponded to a 9% reduction in the risk of chest pain (OR = 0.91, 95%CI: 0.85–0.96, P<0.001). The RCS curve revealed a nonlinear relationship between log₂(STB) and chest pain (P for nonlinearity = 0.008), with an inflection point at 14.42 μmol/L. The association between STB and chest pain was consistent across multiple subgroups.

Mildly elevated STB concentrations were associated with a lower prevalence of chest pain in our study population, suggesting that STB may have potential value for risk stratification of chest pain. Nevertheless, additional prospective studies are required to verify the causal relationship between them.

**Keywords:** chest pain; bilirubin; obesity; cardiovascular disease

**1. Introduction**

Chest pain is defined as discomfort and/or a sense of oppression originating from the sternum and its surrounding areas. In the United States, chest pain ranks as the second leading cause of emergency department visits^1^.Each year, more than 8 million patients with chest pain receive outpatient evaluation, and approximately one-quarter of patients presenting with chest pain require hospital admission for observation.^2,3^. Chest pain has an extensive differential diagnosis covering respiratory, musculoskeletal, psychological, gastric and esophageal disorders, among which cardiovascular diseases are the most life-threatening, including coronary heart disease (CHD), hypertension, angina pectoris and heart failure. Cardiovascular diseases carry fatal potential risks, and chest pain serves as the most typical warning sign, especially for CHD. Meanwhile, the occurrence of chest pain can predict cardiovascular events^4^. Moreover, chest pain exacerbates the burden of depression, and a bidirectional relationship exists between depression and chest pain, which severely impairs physical function and quality of life in patients with chest pain^5,6^.Therefore, identifying influencing factors for chest pain is of great clinical significance.

STB is the final product of heme catabolism by heme oxygenase during erythrocyte senescence and is one of the most potent endogenous antioxidants, which has been confirmed to be inversely associated with the risks of various diseases. Mildly elevated STB correlates with lower cardiovascular disease risk, particularly coronary artery disease. Meanwhile, STB exerts potential protective effects against cardiovascular risk factors, including non-alcoholic fatty liver disease^7^、stroke^8^, peripheral arterial disease^9^, diabetes mellitus and its complications (retinopathy, microalbuminuria)^10-12^. Moreover, multiple studies have found relationships between bilirubin and atrial fibrillation^13^, pulmonary embolism^14^, herpes zoster^15^, anxiety and depression. It is widely recognized that chest pain is a common symptom of these diseases.

In summary, the biological functions of bilirubin have been gradually elucidated, and regulating bilirubin levels to prevent and treat chronic diseases has become a popular research topic, which includes multiple disorders that can induce chest pain. On this basis, we hypothesized that STB, which mirrors systemic inflammatory and oxidative stress burden, is significantly associated with the risk of developing chest pain. The present study was designed to verify this potential relationship, compare the incidence of chest pain across populations with varying STB concentrations, and furnish evidence supporting clinical prevention, treatment and health management for chest pain.

**2 Materials and methods**

#### 2.1 Ethical approval declarations

NHANES is conducted by the Centers for Disease Control and Prevention (CDC) to evaluate the health status of the US population. Participants are recruited nationwide to guarantee sample representativeness. The NHANES protocol was approved by the Research Ethics Review Board of the National Center for Health Statistics of the United States, and written informed consent was obtained from all participants.

### 2.2 Study population

Initially, 70,190 individuals from the NHANES 2005–2018 cycles were enrolled in this study. We excluded participants younger than 20 years old, those with incomplete chest pain diagnostic data, and individuals missing covariate information. Notably, severe liver dysfunction can alter STB concentrations and independently affect the risk of chest pain, thereby leading to reverse causation and disease-related confounding factors. In accordance with previous studies, subjects with liver dysfunction (defined as aspartate aminotransferase (AST) >100 U/L, alanine aminotransferase (ALT) >100 U/L, gamma-glutamyl transferase (GGT) >100 U/L, or STB >51.3 μmol/L) and participants with self-reported liver disease were excluded^16^. Finally, a total of 19,187 participants were included for subsequent analysis (Figure 1).

**Figure 1:**Inclusion and exclusion flow diagram

STB: Serum Total Bilirubin

### 2.3 Exposure and Outcome

STB concentrations were measured via the timed endpoint diazo method (Jendrassik–Grof method), and STB levels were recorded in μmol/L in the NHANES database. The primary outcome was the presence or absence of chest pain. Standardized Rose Angina Questionnaire was used by investigators to assess chest pain symptoms of participants. Subjects who answered affirmatively to the question “Have you experienced any pain or discomfort in your chest?” were classified as having chest pain; otherwise, they were categorized into the non-chest pain group^17^。

### 2.4 Covariates

Covariates incorporated in this study covered age, marital status, race, smoking status, BMI, educational attainment, diabetes mellitus, hypertension, chronic kidney disease (CKD), heart failure, CHD, myocardial infarction, angina, stroke, asthma and depression, as well as laboratory indicators including high-density lipoprotein (HDL), total cholesterol, lactate dehydrogenase (LDH), AST, ALT and GGT.Smoking status was divided into three groups (current smoker, former smoker, never smoker) based on two questionnaire items: “Have you smoked at least 100 cigarettes in your entire life?” and “Do you currently smoke cigarettes?”.The CKD Epidemiology Collaboration (CKD-EPI) formula was adopted to calculate estimated glomerular filtration rate (eGFR). CKD was defined as urinary albumin-to-creatinine ratio (ACR) ≥ 30 mg/g or eGFR < 60 mL/min/1.73 m²^18,19^ .Diabetes was diagnosed according to self-reported physician-diagnosed diabetes, insulin or hypoglycemic medication use, fasting blood glucose ≥ 126 mg/dL, or glycated hemoglobin ≥ 6.5%. Hypertension was defined as physician-diagnosed hypertension or average systolic blood pressure ≥ 140 mmHg / average diastolic blood pressure > 90 mmHg.Marital status (cohabiting/living alone), asthma, cardiovascular disease, stroke, malignancy and depression were all determined by self-reported questionnaires. Cardiovascular disease encompassed CHD, angina, heart failure and prior cardiovascular events (myocardial infarction).

### 2.5 Statistical analyses

Continuous variables were presented as mean ± standard deviation, while categorical variables were expressed as percentages. Differences between groups were compared using the t-test or chi-square test accordingly. Three sequentially adjusted logistic regression models were constructed to examine the association between STB and chest pain, with adjusted odds ratios (ORs) and 95% confidence intervals (95% CIs) reported. Restricted cubic spline (RCS) analysis was performed to explore the nonlinear relationship between STB and chest pain. Model 1 was unadjusted. Model 2 was adjusted for age, gender, BMI, marital status, educational level, race, and smoking status. Model 3 was further adjusted for CKD, diabetes mellitus, hypertension, heart failure, CHD, myocardial infarction, angina, stroke, asthma, depression, HDL, total cholesterol, LDH, AST, ALT, and GGT on the basis of Model 2. In addition, logistic regression and RCS analyses were repeated in two supplementary cohorts: 21,010 participants including individuals with liver dysfunction and self-reported liver disease, and participants without cardiovascular diseases. All statistical analyses were conducted using R software (version 4.5). Two-tailed P < 0.05 was considered statistically significant.

**3. Results**

### 3.1. Characteristics of the participants

A total of 19,187 adults were enrolled in the present study, and Table 1 summarizes the baseline characteristics stratified by chest pain status (chest pain vs. no chest pain). Males accounted for 48.5% and females for 51.5%, with no statistically significant difference in gender distribution between the two groups (P = 0.086). The overall mean age was 59.83 ± 12.25 years. Compared with participants without chest pain, individuals experiencing chest pain were older and had higher BMI, LDH and GGT levels, accompanied by lower levels of HDL, total cholesterol, STB, AST and ALT. Subjects with chest pain exhibited higher prevalence rates of heart failure, angina, CHD, myocardial infarction, diabetes mellitus, hypertension, stroke, cancer, asthma, CKD and depression. Significant intergroup differences were also observed in smoking status, marital status and race, whereas educational attainment showed no statistical difference. Detailed data are presented in Table 1. Baseline characteristics of the 21,010 participants including those with liver dysfunction and self-reported liver disease are provided in Supplementary Table 1.

**Table1:**Baseline characteristics table

STB：serum total bilirubin;AST：Aspartate Aminotransferase;ALT：Alanine Aminotransferase;GGT：Gamma-Glutamyl Transferase;LDH：Lactate Dehydrogenase;HDL：High-Density Lipoprotein;TC：Total Cholesterol;CKD：Chronic Kidney Disease;CHD：Coronary Heart Disease

### 3.2. Association between STB and chest pain

The associations between STB and chest pain risk are summarized in Table 2. In Model 1 without covariate adjustment, log(STB) was negatively correlated with chest pain (OR = 0.87, 95% CI: 0.82–0.91, P < 0.001). After adjusting for demographic variables in Model 2 (OR = 0.90, 95% CI: 0.86–0.95, P < 0.001) and full covariate adjustment in Model 3 (OR = 0.91, 95% CI: 0.85–0.96, P < 0.001), the association between STB and chest pain remained statistically significant. In Model 3, compared with quartile 1 (Q1), the ORs and corresponding 95% CIs for the correlation between quartile 2 (Q2), quartile 3 (Q3), quartile 4 (Q4) and chest pain were 0.92 (0.82, 1.03), 0.87 (0.79, 0.96), and 0.86 (0.78, 0.95), respectively.

**Table 2**:Association between STB and chest pain

Model 1: unadjusted

Model 2: adjusted for age, BMI, sex, marital status, educational level, race and smoking status

Model 3: adjusted for all covariates included in Model 2 plus CKD, hypertension, diabetes mellitus, CHD, angina pectoris, heart failure, myocardial infarction, stroke, asthma, cancer, depression, total cholesterol, HDL, LDH, AST, ALT and GGT

### 3.3. RCS Curve for the Association between STB and Chest Pain

The RCS curve demonstrated a nonlinear association between STB and the risk of chest pain (P for nonlinearity = 0.008) (Figure 2). A threshold value of 14.42 μmol/L was identified. When STB was below 14.42 μmol/L, elevated STB was associated with a reduced risk of chest pain (OR = 0.91, 95% CI: 0.86–0.96). No significant correlation was observed between STB and chest pain risk when STB was ≥ 14.42 μmol/L (OR = 1.21, 95% CI: 0.90–1.62).

Figure 2：RCS curve depicting the relationship between STB and chest pain risk

Smooth Curve Fitting Plot for the Dose-Response Relationship between STB and Chest Pain; Adjusted for Age, gender, BMI, Marital Status, Educational Level, Ethnicity, Smoking Status, CKD, Hypertension, Diabetes Mellitus, CHD, Angina Pectoris, Heart Failure, Myocardial Infarction, Stroke, Asthma, Cancer, Depression, Total Cholesterol, HDL, LDH, AST, ALT and GGT;

### 3.4. Subgroup analyses

The results of subgroup interaction analyses are presented in Figure 3. The significant association between STB and chest pain persisted in females, obese individuals, participants without hypertension, diabetes, stroke, CKD, malignancy, asthma or depression, as well as current and former smokers. Notably, a significant interaction was detected between normal/overweight weight status and obesity subgroups (P for interaction = 0.043). STB was significantly negatively correlated with chest pain in the obese subgroup, whereas no statistically significant association was found in the normal-weight and overweight subgroups.

Figure3:Subgroup analyses

Forest plots were used for subgroup analysis; CKD: Chronic Kidney Disease; normal weight and overweight were defined as a BMI < 30; obesity was defined as a BMI ≥ 30; STB: Serum Total Bilirubin.

### 3.5. Sensitivity Analysis

We re‑analyzed three supplementary analyses using logistic regression and RCS curves. The first analysis comprised 21,010 participants, including subjects with liver dysfunction and self‑reported liver disease (Supplementary Table 1, Supplementary Table 2, Supplementary Figure 1). The second analysis included 16,952 participants after excluding individuals with cardiovascular diseases (Supplementary Table 3, Supplementary Figure 2). The third analysis evaluated the association between STB and chest‑pain risk by incorporating daily alcohol intake into the model (Supplementary Table 4, Supplementary Table 5, Supplementary Figure 3, Supplementary Figure 4). Findings from all three supplementary analyses were consistent with those from the primary analysis, further supporting our conclusions.

**4. Discussion**

For the first time, we explored the association between STB and chest pain symptoms based on a large sample size and identified an inverse dose–response relationship between chest pain and STB concentrations in our study population. Threshold effect analysis further revealed that STB levels below 14.42 μmol/L were linked to a lower incidence of chest pain, whereas no significant correlation existed when STB was ≥ 14.42 μmol/L. These findings may carry important implications for risk assessment of chest pain.

Existing studies have verified a potential link between STB and diseases manifesting with chest pain. In a prospective cohort of 7,222 participants, Setor K. Kunutsor et al. found that each standard deviation increment in STB was associated with an 11% reduction in cardiovascular disease risk. Their meta-analysis further demonstrated that per standard deviation rise in STB reduced the overall risk of cardiovascular disease by 7% and the risk of CHD by 5%^20^. Seung Joo Kang et al. reported that patients with hyperbilirubinemia (>1.2 mg/dL) had a markedly lower prevalence of atherosclerotic stenosis >50% and calcified plaques compared with those in the low-bilirubin group (≤1.2 mg/dL)^21^. Hakan Gullu et al. confirmed that higher physiological STB concentrations were associated with superior coronary flow reserve in healthy young adults, indicating a potential protective effect of STB against coronary microvascular injury and atherosclerosis^22^.Another study revealed that elevated STB was associated with favorable collateral vessel development in patients with chronic total coronary occlusion^23^.A cross-sectional study observed significantly lower STB concentrations among patients with atrial fibrillation relative to controls, and the anti-inflammatory and antioxidant properties of bilirubin were proposed as the underlying mechanism^24^.Another investigation suggested that direct bilirubin might serve as a potential predictor for pulmonary embolism following hip surgery^25^. A case–control study indicated that individuals with STB <8.6 μmol/L exhibited a 73% higher risk of pulmonary embolism relative to controls^26^. Tuba Oskay et al. discovered reduced STB levels in patients with herpes zoster versus healthy controls^27^.whereas another study showed that subjects with STB ≥9.2 μmol/L had a 69.8% lower risk of developing postherpetic neuralgia^28^。

Our findings are largely consistent with previous literature. Logistic regression and subgroup analyses were performed to explore the relationship between STB and chest pain in the current study. Our results indicated that elevated STB was inversely correlated with the prevalence of chest pain. Subgroup analyses identified a significant difference in the STB–chest pain association between obese individuals and those with normal or overweight body weight; the correlation was stronger within the obese population, which may suggest a protective effect of STB against obesity and obesity-related metabolic disorders^29,30^. Nevertheless, owing to the multiple subgroup analyses conducted and the lack of definitive mechanistic explanations, this observation requires further validation.

Several plausible mechanisms may explain the association between STB and chest pain. Given the anti-inflammatory and antioxidant properties of STB, higher STB concentrations may improve coronary endothelial function by eliminating reactive oxygen species and enhancing nitric oxide bioavailability^22,31-33^.Meanwhile, Patrick F McArdle et al. demonstrated that the UGT1A1*28 genotype, a key genetic variant governing bilirubin metabolism, was correlated with brachial artery diameter and cold pressor reactivity, implying that STB exerts early regulatory effects on vascular structure and vascular responsiveness^34^. Second, prior research has confirmed that oxidative stress and inflammatory markers (such as albumin-to-red cell distribution width ratio and neutrophil-to-albumin ratio) are related to the incidence and prognosis of chest pain, and systemic oxidative stress and inflammatory burden can promote the development of chest pain. Third, under oxidative stress, activated platelets aggregate to trigger thrombosis while exacerbating inflammation and endothelial dysfunction; the antioxidant capacity of STB may contribute to regulating platelet function in vivo^35-37^. In addition, as a biomarker of inflammation and oxidative stress, STB has been found to correlate with psychiatric disorders^38,39^, pulmonary embolism^40,41^ and herpes zoster^15,28^, suggesting that STB may be involved in multiple etiologies responsible for chest pain. However, precise biological mechanisms remain to be clarified through additional experimental and longitudinal studies.

Notably, albumin also participates in the pathogenesis of chest pain and possesses long-term prognostic predictive value^42-44^.Sulfhydryl groups within albumin scavenge reactive oxygen species, inhibit lipid peroxidation, and suppress the secretion of pro-inflammatory factors including TNF-α and IL-6, thereby protecting blood vessels and stabilizing atherosclerotic plaques^45^. Hypoalbuminemia-induced malnutrition exacerbates systemic frailty and consequently increases the risk of chest pain^46,47^ and albumin also carries independent prognostic value^42^.STB reflects systemic oxidative stress status, while albumin reflects systemic anti-inflammatory, antioxidant capacity and nutritional reserves. Multiple studies have reported associations between plasma bilirubin levels and all-cause mortality^48,49^, implying that bilirubin and albumin may share overlapping functional pathways.

The RCS curves revealed a nonlinear relationship between STB and chest pain, and segmented regression identified a distinct inflection point at 14.42 μmol/L. STB was negatively associated with chest pain below this threshold, whereas no significant correlation was observed above 14.42 μmol/L. Sensitivity analyses yielded results consistent with the primary analyses. This nonlinear pattern is also observed in the associations between STB and other chronic diseases[^50-52^. A possible explanation is that once STB exceeds this threshold, its antioxidant property shifts toward cytotoxicity^53^。

The present study had a sufficient sample size, and multiple potential confounding factors were adjusted for during statistical analyses. The consistent results across subgroup analyses and sensitivity analyses guaranteed robust conclusions. Nevertheless, several limitations should be acknowledged.First, the chest pain questionnaire adopted by NHANES cannot distinguish the type and nature of chest pain, making it impossible to clarify the associations between STB and different subtypes of chest pain. Although the overall sample size was large, participants with missing data were excluded during analysis. Furthermore, covariates such as alcohol consumption and physical activity were excluded from the regression models due to high missing rates, which might introduce selection bias.Second, substantial missing data existed in the Rose questionnaire chest pain module within the NHANES database, so chest pain was defined by only a single item in this study. Although this definition has been validated in other large cohort studies, recall bias and misunderstanding of questionnaire items among participants may lead to misclassification of the outcome.Third, STB was not stratified in our analysis; we failed to explore the separate relationships of direct bilirubin and indirect bilirubin with chest pain. Targeted research focusing on these two fractions of bilirubin can be carried out in future work.Fourth, Gilbert syndrome, also known as benign hyperbilirubinemia, may serve as a critical confounder in this study. Patients with Gilbert syndrome may benefit from mild hyperbilirubinemia, including reduced risks of cardiovascular diseases and other metabolic disorders^54,55^.Restricted by data availability of the public database, we could not identify or adjust for individuals with Gilbert syndrome, and omission of this confounder may distort the effect size between STB and chest pain.Finally, although our results demonstrated that higher bilirubin levels were correlated with lower chest pain risk, it must be emphasized that causal inference cannot be drawn from observational data. As a cross-sectional study, this research only explored associations rather than causal relationships. It remains unclear whether abnormal bilirubin levels trigger chest pain or whether chest pain-related lesions alter bilirubin metabolism. Rigorous experimental studies and longitudinal cohorts are required to determine causality in the future.

**5. Conclusion**

In our study population, elevated STB was associated with a lower risk of chest pain, accompanied by a significant nonlinear relationship and threshold effect at an STB concentration of 14.42 μmol/L. Below this cutoff value, bilirubin was inversely correlated with chest pain risk, indicating that bilirubin may have potential applications in chest pain risk stratification. Nevertheless, owing to the inherent limitations of the chest pain questionnaire in the NHANES database, chest pain was not classified as cardiac or non-cardiac, intermittent or persistent. Hence, our findings should be interpreted cautiously. Comprehensive investigations into the relationship between STB and distinct chest pain subtypes are warranted in future research. Additionally, further prospective studies are needed to establish the causal relationship between bilirubin and chest pain.

**Author contributions**

All authors were involved in the conception and design of this study. Among them,Z. F., Z. W., J. Y., Y. H. J. T and C.Y.were responsible for data collection and analysis. The initial draft was written by J. Z., and M. Q. provided feedback on multiple revisions of the manuscript. The final version was reviewed and approved by all authors.

**Data availability**

The datasets supporting the conclusions of this article are available in the [NHANES](https://www.cdc.gov/nchs/nhanes/) database repository.（<https://www.cdc.gov/nchs/nhanes/>）

**Competing interests**

The authors declare no competing interests.

**Funding**

This research received no specific grant from any funding agency in the public, commercial, or not-for-profit sectors.

**References:**

1. Lenfant C. Chest pain of cardiac and noncardiac origin. *Metabolism*. 2010;59 Suppl 1:S41-6

2. Gulati M, Levy PD, Mukherjee D et al. 2021 AHA/ACC/ASE/CHEST/SAEM/SCCT/SCMR Guideline for the Evaluation and Diagnosis of Chest Pain: A Report of the American College of Cardiology/American Heart Association Joint Committee on Clinical Practice Guidelines. *Circulation*. 2021;144(22):e368-e454

3. Safdar B, Dziura J, Bathulapalli H et al. Chest pain syndromes are associated with high rates of recidivism and costs in young United States Veterans. *BMC Fam Pract*. 2015;16:88

4. Robson J, Ayerbe L, Mathur R, Addo J, Wragg A. Clinical value of chest pain presentation and prodromes on the assessment of cardiovascular disease: a cohort study. *BMJ Open*. 2015;5(4):e007251

5. Jespersen L, Abildstrom SZ, Hvelplund A, Prescott E. Persistent angina: highly prevalent and associated with long-term anxiety, depression, low physical functioning, and quality of life in stable angina pectoris. *Clin Res Cardiol*. 2013;102(8):571-81

6. Sara J, Rajai N, Breitinger S et al. Mental stress is associated with coronary endothelial dysfunction in women with chest pain and non-obstructive coronary artery disease. *Int J Cardiol Heart Vasc*. 2025;61:101802

7. Han H, Yu Q, Qin N et al. Non-linear associations of circulating total bilirubin concentration with the risk of nonalcoholic fatty liver disease and all-cause mortality. *Ann Hepatol*. 2024;29(2):101177

8. Wang X, Wu D, Zhong P. Serum bilirubin and ischaemic stroke: a review of literature. *Stroke Vasc Neurol*. 2020;5(2):198-204

9. Perlstein TS, Pande RL, Beckman JA, Creager MA. Serum total bilirubin level and prevalent lower-extremity peripheral arterial disease: National Health and Nutrition Examination Survey (NHANES) 1999 to 2004. *Arterioscler Thromb Vasc Biol*. 2008;28(1):166-72

10. Yang M, Ni C, Chang B et al. Association between serum total bilirubin levels and the risk of type 2 diabetes mellitus. *Diabetes Res Clin Pract*. 2019;152:23-28

11. Yasuda M, Kiyohara Y, Wang JJ et al. High serum bilirubin levels and diabetic retinopathy: the Hisayama Study. *Ophthalmology*. 2011;118(7):1423-8

12. Fukui M, Tanaka M, Shiraishi E et al. Relationship between serum bilirubin and albuminuria in patients with type 2 diabetes. *Kidney Int*. 2008;74(9):1197-201

13. Liang H, Zhang S, Zhu Y. Association between blood test indicators and atrial fibrillation in elderly patients aged 65 and above in the Central Jiangsu region: a cross-sectional study. *BMC Cardiovasc Disord*. 2025;25(1):51

14. Cui M, Wu Y, Tong S et al. Risk factors of pulmonary embolism in postpartum women. *Front Med (Lausanne)*. 2025;12:1617052

15. Oskay T, Keskin C, Özen M. Antioxidant and inflammatory biomarkers in herpes zoster. *J Med Virol*. 2022;94(8):3924-3929

16. Ong KL, Allison MA, Cheung BM et al. The relationship between total bilirubin levels and total mortality in older adults: the United States National Health and Nutrition Examination Survey (NHANES) 1999-2004. *PLoS One*. 2014;9(4):e94479

17. Zhao Y, Gu Y, Zhang B. Associations of triglyceride-glucose (TyG) index with chest pain incidence and mortality among the U.S. population. *Cardiovasc Diabetol*. 2024;23(1):111

18. Kang H, Lee JP, Choi K. Exposure to phthalates and environmental phenols in association with chronic kidney disease (CKD) among the general US population participating in multi-cycle NHANES (2005-2016). *Sci Total Environ*. 2021;791:148343

19. Sumida K, Nadkarni GN, Grams ME et al. Conversion of Urine Protein-Creatinine Ratio or Urine Dipstick Protein to Urine Albumin-Creatinine Ratio for Use in Chronic Kidney Disease Screening and Prognosis : An Individual Participant-Based Meta-analysis. *Ann Intern Med*. 2020;173(6):426-435

20. Kunutsor SK, Bakker SJ, Gansevoort RT, Chowdhury R, Dullaart RP. Circulating total bilirubin and risk of incident cardiovascular disease in the general population. *Arterioscler Thromb Vasc Biol*. 2015;35(3):716-24

21. Kang SJ, Kim D, Park HE et al. Elevated serum bilirubin levels are inversely associated with coronary artery atherosclerosis. *Atherosclerosis*. 2013;230(2):242-8

22. Gullu H, Erdogan D, Tok D et al. High serum bilirubin concentrations preserve coronary flow reserve and coronary microvascular functions. *Arterioscler Thromb Vasc Biol*. 2005;25(11):2289-94

23. Erdogan T, Cicek Y, Kocaman SA et al. Increased serum bilirubin level is related to good collateral development in patients with chronic total coronary occlusion. *Intern Med*. 2012;51(3):249-55

24. Demir M, Demir C, Uyan U, Melek M. The Relationship Between Serum Bilirubin Concentration and Atrial Fibrillation. *Cardiol Res*. 2013;4(6):186-191

25. Ding R, Ding Y, Zheng D et al. Machine Learning-Based Screening of Risk Factors and Prediction of Deep Vein Thrombosis and Pulmonary Embolism After Hip Arthroplasty. *Clinical and applied thrombosis/hemostasis : official journal of the International Academy of Clinical and Applied Thrombosis/Hemostasis*. 2023;29:10760296231186145

26. Cervellin G, Buonocore R, Sanchis-Gomar F, Lippi G. Low serum bilirubin values are associated with pulmonary embolism in a case-control study. *Clin Chem Lab Med*. 2016;54(8):e229-e230

27. Oskay T, Keskin C, Zen M. Antioxidant and inflammatory biomarkers in herpes zoster. *J Med Virol*. 2022;94(8):3924-3929

28. Yue J, Yao M. Predictive Value of Preoperative Antioxidant Levels in Postherpetic Neuralgia: A Retrospective Study. *Pain Physician*. 2025;28(5):431-438

29. Tian L, Zhang JG, Chen Y et al. A Cross-sectional Data Analysis between Serum Total Bilirubin and Potential Obesity Indices in US Adults. *Curr Mol Med*. 2025;

30. Vítek L. Bilirubin as a signaling molecule. *Med Res Rev*. 2020;40(4):1335-1351

31. Maruhashi T, Kihara Y, Higashi Y. Bilirubin and Endothelial Function. *J Atheroscler Thromb*. 2019;26(8):688-696

32. Yoshino S, Hamasaki S, Ishida S et al. Characterization of the effect of serum bilirubin concentrations on coronary endothelial function via measurement of high-sensitivity C-reactive protein and high-density lipoprotein cholesterol. *Heart Vessels*. 2013;28(2):157-65

33. Quyyumi AA, Cannon RR, Panza JA, Diodati JG, Epstein SE. Endothelial dysfunction in patients with chest pain and normal coronary arteries. *Circulation*. 1992;86(6):1864-71

34. McArdle PF, Whitcomb BW, Tanner K et al. Association between bilirubin and cardiovascular disease risk factors: using Mendelian randomization to assess causal inference. *BMC Cardiovasc Disord*. 2012;12:16

35. Kundur AR, Singh I, Bulmer AC. Bilirubin, platelet activation and heart disease: a missing link to cardiovascular protection in Gilbert's syndrome? *Atherosclerosis*. 2015;239(1):73-84

36. Lindenblatt N, Bordel R, Schareck W, Menger MD, Vollmar B. Vascular heme oxygenase-1 induction suppresses microvascular thrombus formation in vivo. *Arteriosclerosis, thrombosis, and vascular biology*. 2004;24(3):601-606

37. Duman H, Zyurt S, Erdo An T, Kara BYL, Durako Lugil ME. The role of serum bilirubin levels in determining venous thromboembolism. *Journal of vascular surgery. Venous and lymphatic disorders*. 2019;7(5):635-639

38. Liu Y, Wang Z, Li D, Lv B. Bilirubin and postpartum depression: an observational and Mendelian randomization study. *Front Psychiatry*. 2024;15:1277415-1277415

39. Lu Z, Pu C, Zhang Y et al. Oxidative Stress and Psychiatric Disorders: Evidence from the Bidirectional Mendelian Randomization Study. *Antioxidants (Basel, Switzerland)*. 2022;11(7):1386

40. Liu Q, Xiao J, Liu L et al. A new nomogram prediction model for pulmonary embolism in older hospitalized patients. *Heliyon*. 2024;10(3):e25317-e25317

41. Cervellin G, Buonocore R, Sanchis-Gomar F, Lippi G. Low serum bilirubin values are associated with pulmonary embolism in a case-control study. *Clin Chem Lab Med*. 2016;54(8):e229-e230

42. Hay Ro Lu ML, Nar T, Inier GK et al. Prognostic value of serum albumin for long-term mortality in patients with dual-chamber permanent pacemakers. *Biomark Med*. 2022;16(5):341-348

43. Zhu M, Huang D, Xi H. The association between red blood cell distribution width-to-albumin ratio and chest pain prevalence in US adults: A cross-sectional analysis of NHANES 2005-2018. *Medicine (Baltimore)*. 2025;104(27):e43200

44. Long Q, Liu Z, Guo Q, Song X, Guo Y. Association between neutrophil percentage-to-albumin ratio (NPAR) and long-term mortality in community-dwelling adults with chest pain: Evidence from US NHANES 2001-2018. *Clinica e investigacion en arteriosclerosis : publicacion oficial de la Sociedad Espanola de Arteriosclerosis*. 2026;38(3):500843

45. Hay Ro Lu ML, Altay S. Overlap Between Nutritional Indices in Patients with Acute Coronary Syndrome: A Focus on Albumin. *Balkan Med J*. 2024;41(5):324-325

46. Zhang Z, Chen M, Cai D et al. Association between frailty and chest pain: Insights from the 2009 to 2018 NHANES cross-sectional analysis and Mendelian randomization. *Medicine (Baltimore)*. 2025;104(38):e44517

47. Sun L, Li C, Feng B et al. The Associations between Albumin, Globulin, Albumin-to-globulin Ratio, and Frailty in Middle-aged and Older Adults: Evidence from NHANES 2013-2014. *Curr Pharm Des*. 2025;31(41):3303-3311

48. Moss K, Dennis BB, Naji L, Ahmed A, Kim D. Total Bilirubin Levels in Nonalcoholic Fatty Liver Disease and All- cause and Cause-specific Mortality in US Adults. *Journal of gastrointestinal and liver diseases : JGLD*. 2023;32(3):323-331

49. Vitek L, Hubacek JA, Pajak A et al. Association between plasma bilirubin and mortality. *Ann Hepatol*. 2019;18(2):379-385

50. Liu M, Wang J, He Y. The U-Shaped Association between Bilirubin and Diabetic Retinopathy Risk: A Five-Year Cohort Based on 5323 Male Diabetic Patients. *J Diabetes Res*. 2018;2018:4603087

51. Liu Y, Wang J, Zeng WZ, Lyu QS. Nonlinear relationship between serum total bilirubin levels and initial ischemic stroke in patients with non-valvular atrial fibrillation. *J Int Med Res*. 2020;48(10):300060520962347

52. Jiang B, Ling C, Dong B, Lu X, Liu Y. Associations between serum total bilirubin and overactive bladder from the National Health and Nutrition Examination Survey. *Front Endocrinol (Lausanne)*. 2024;15:1421426

53. Bianco A, Dvorak A, Capkova N et al. The Extent of Intracellular Accumulation of Bilirubin Determines Its Anti- or Pro-Oxidant Effect. *Int J Mol Sci*. 2020;21(21)

54. Vitek L, Tiribelli C. Gilbert's syndrome revisited. *J Hepatol*. 2023;79(4):1049-1055

55. Lin J, O'Donnell CJ, Schwaiger JP et al. Association between the UGT1A1*28 allele, bilirubin levels, and coronary heart disease in the Framingham Heart Study. *Circulation*. 2006;114(14):1476-1481

**Table1:**Baseline characteristics of participants

**Table 2**:Association between STB and chest pain

**Figure 1:**Flow diagram of the inclusion criteria and exclusion criteria

**Figure 2:**RCS curve depicting the association between STB and chest pain risk

**Figure 3:**Forest plots depicting subgroup analysis of STB and chest‑pain risk

**Supplementary**

**Supplementary Table 1**: Baseline characteristics table for analysis incorporating participants with abnormal liver function

**Supplementary Table 2**: Association between STB and chest pain when incorporating participants with abnormal liver function

**Supplementary Table 3**: Association between STB and chest pain after excluding participants with cardiovascular diseases

**Supplementary Table 4：**Baseline characteristics table excluding participants missing alcohol intake data

**Supplementary Table 5：**Association between STB and Chest Pain excluding participants missing alcohol intake data

**Supplementary Figure 1：**RCS curve depicting the relationship between STB and chest pain risk when incorporating participants with abnormal liver function

**Supplementary Figure 2：**RCS curve depicting the relationship between STB and chest pain risk after excluding participants with cardiovascular diseases

**Supplementary Figure 3:**Inclusion and exclusion flow diagram excluding participants missing alcohol intake data

**Supplementary Figure 4：**RCS curve depicting the relationship between STB and chest pain risk excluding participants missing alcohol intake data
